# Analyzing Self-Similar and Fractal Properties of the *C. elegans* Neural Network

**DOI:** 10.1371/journal.pone.0040483

**Published:** 2012-10-05

**Authors:** Tyler M. Reese, Antoni Brzoska, Dylan T. Yott, Daniel J. Kelleher

**Affiliations:** 1 Department of Mathematics, University of Connecticut, Storrs, Connecticut, United States of America; 2 Department of Mathematics, Boston University, Boston, Massachusetts, United States of America; Institute of Psychology, Chinese Academy of Sciences, China

## Abstract

The brain is one of the most studied and highly complex systems in the biological world. While much research has concentrated on studying the brain directly, our focus is the structure of the brain itself: at its core an interconnected network of nodes (neurons). A better understanding of the structural connectivity of the brain should elucidate some of its functional properties. In this paper we analyze the connectome of the nematode *Caenorhabditis elegans*. Consisting of only 302 neurons, it is one of the better-understood neural networks. Using a Laplacian Matrix of the 279-neuron “giant component” of the network, we use an eigenvalue counting function to look for fractal-like self similarity. This matrix representation is also used to plot visualizations of the neural network in eigenfunction coordinates. Small-world properties of the system are examined, including average path length and clustering coefficient. We test for localization of eigenfunctions, using graph energy and spacial variance on these functions. To better understand results, all calculations are also performed on random networks, branching trees, and known fractals, as well as fractals which have been “rewired” to have small-world properties. We propose algorithms for generating Laplacian matrices of each of these graphs.

## Introduction

Fractal theory has become an increasingly prevalent topic of both debate and research in recent years. Beginning with Mandelbrot's discussion of Britain's immeasurable coastline [1], fractal analysis has found applications in both the mathematics and scientific communities. In the geometric sense, fractals are objects that contain self-symmetry: they exhibit the same pattern on increasingly smaller scales.

More recently, fractal theory has found applications in the biological realm. Kinetics of ion channels have been modeled with fractal structures [2], [3]. Fractal dimension has been used to analyze human EEG signals [4] as well as the complex morphology of living cells [5], [6]. The applications of fractal theory in neuroscience have been a particularly prevalent topic of research [7]–[9]. Glial cells have been analyzed in-depth using fractal dimensions and modeling [10]–[12]. Dendritic branching has been shown to exhibit self-similarity [13], [14], and three-dimensional fractal structures have been used to approximate the white matter surface of the human brain, based on MRI images [15]. Nevertheless, some have warned against the possible misuses of fractal theory in neuroscience [16], [17]. In particular, calculations on fractal dimensions of biological systems have been called into question, where some studies have attempted to use this measurement as an overly-generalized tool which lacks definite relation to actual biological mechanisms.

In this paper we use a graph-theoretical approach to probe the structure of the *Caenorhabditis elegans* neural network for self-similarity. Similar work was done by Sporns in examining the presence of fractal patterns in neuron connectivity: in [18], fractal networks were generated and structural measures were calculated, including small-world properties, complexity, and motif composition. Advances in graph theory have proven useful in analyzing complex neural networks [19]–[21] as well as underlying motifs in the brain [22], [23]. In this paper we apply mathematical techniques to a physical map of the *C. elegans* connectome. With a well-connected component of only 279 neurons, it is an excellent candidate for graph theoretical research on a complete-brain model. While [24] presents a geometric structure of this system, our research builds upon that of [25] in which Varshney et al. propose a finalized schematic of the *C. elegans* neural network.

The *C. elegans* brain is composed of three types of neuronal cells: sensory neurons, motor neurons, and interneurons. Two types of connection exist between these neurons: chemical synapses and gap junctions. The gap junction network, which sends electrical signals via ion transport, is an undirected system. Conversely, chemical synapses possess clear directionality [25]. In this paper we study the overarching connectivity between neurons. In order to analyze the fundamental network-structure of the *C. elegans* neurons, we consider only the skeleton of the brain's organization. Although some neurons share multiple points of contact and chemical synapses send directional signals, we only observe that two neurons are connected. As a result, we study an undirected and unweighted network combining the chemical and gap junctions, representing only the framework of connections (See Methods). In the process, this analysis loses many of the biological details which correspond to functionality and neuron hierarchy. While such information is vital in understanding the mechanisms acting within the brain, the goal of this study is to mathematically analyze the system's structural connectivity, whereby this simplified network is sufficient.

In order to index each of these connections we use the graph Laplacian matrix, 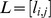. For a graph, 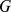, we define 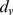 as the degree of vertex 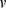: the number of total connections. If vertex 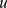 is connected to vertex 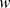 then 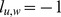 and 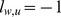, where 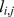 corresponds to the entry in the 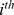 row and 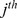 column. Furthermore, 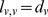, and all other entries of matrix 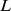 are 0. The original goal of this study was to examine the structure of the *C. elegans* neural network for self-similarity, and results were compared to identical calculations on other graphs. One should note that although fractal theory has repeatedly been applied in neuroscience, in studying the structure of a network the results are not as simple as saying “fractal” or “not-fractal.” Instead we search specifically for *self-similar* structures in the network's organization.

## Results and Discussion

For the *C. elegans* model, we derived a Laplacian matrix from the adjacency matrices used in [25]. Algorithms were developed to produce similar matrices representing random graphs, branching trees, and rewired fractal geometries. These graphs were generated with similar properties as the *C. elegans* neural network, including number of vertices and probability of connection. Details on matrix generation can be found in Methods.

### The Eigenvalue Counting Function

**Figure 1:**
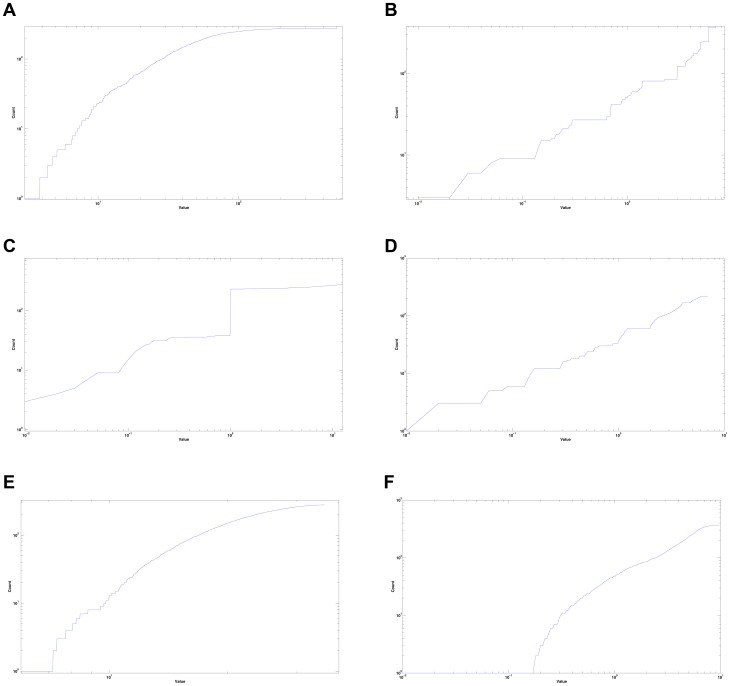
Weyl Ratios (a) *C. elegans* neural network (b) Sierpinski Gasket, Level 5 (c) Random Tree 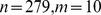 (d) Hexacarpet Level 3 (e) Random Network 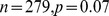 (f) Sierpinski Gasket Rewiring 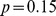.

The eigenvalue counting function is a cumulative distribution function on the spectrum of a matrix, in this case the Laplacian (see Methods). Plotting this function gives an expedient way to analyze the spectrum of the graph Laplacian [26]. It is known that this function exhibits spectral “gaps” when applied to fractal geometries, corresponding to sections of slope-zero in the plot [27]. The asymptotics of this function have also been shown to be linked with heat dissipation in networks [28]. Figure 1 shows plots of the eigenvalue counting function on the Laplacian matrices.

There is a clear presence of step-like portions of those graphs corresponding to known fractals. These sections of slope-zero correspond to spectral gaps, consistent with expected results. The eigenvalue counting function plot of the *C. elegans* connectome (Fig. 1 (a)) does not show definitive spectral gaps, indicating that the nematode brain is *not* strictly fractal in structure. This, however, does not eliminate the possibility of some degree of self-similarity. Figure 1 (c) contains the graph corresponding to a random-branching tree. The large vertical jump at 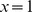, with a change on the y-axis of approximately 200, indicates that the eigenvalue 1 occurs with extremely high multiplicity. This is caused by the nature of the tree's organization. There is a large number of endpoints: vertices at which no further branching occurs, connected only to the “parent” vertex. As the highly interconnected neural network is not tree-like, dissimilarity in observed eigenvalue counting patterns is consistent with expected results. Although the eigenvalue counting function of the *C. elegans* neural network does resemble those of the random network (Fig. 1 (e)) and the rewired Sierpinski Gasket (Fig. 1 (f)), this cannot conclusively point to similar structural organization, whereas drastic dissimilarity would point to fundamental differences.

### Weyl Ratios

The Weyl ratio of a graph is defined as
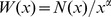
where 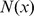 is the eigenvalue counting function. 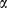 is determined by the logarithmic asymptotics of the eigenvalue counting function. One way to determine this 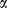 is via a linear regression on 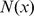 when plotted on logarithmic axes. Log-log periodicity of Weyl ratios is present in fractal geometries, and has been observed in graph-approximations of fractals. Similar periodicity in Weyl ratio patterns of other networks can point to self-similarity in these graphs. For more on Weyl ratio analysis on fractals, see [29].

**Figure 2:**
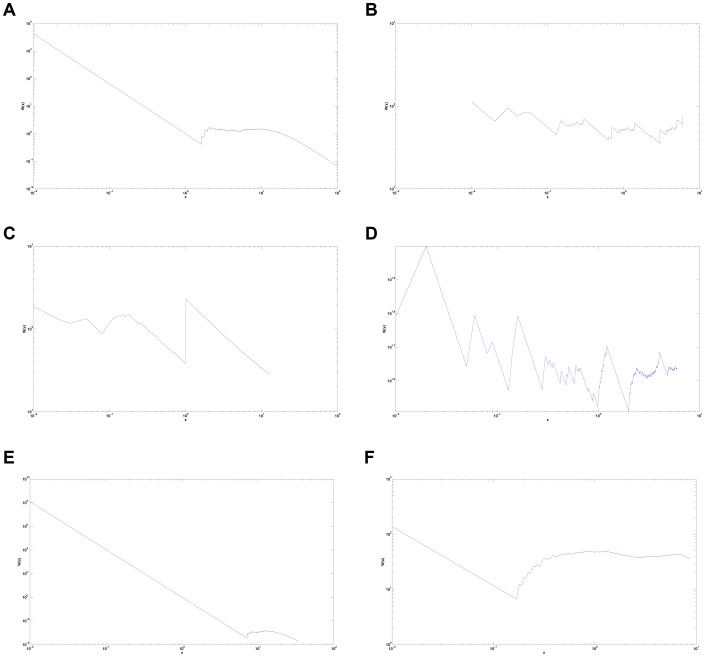
Weyl Ratios (a) *C. elegans* neural network (b) Sierpinski Gasket, Level 5 (c) Random Tree 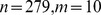 (d) Hexacarpet Level 3 (e) Random Network 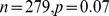 (f) Sierpinski Gasket Rewiring 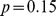.

As expected, the Weyl ratios of known self-similar fractals show a high degree of organization. That of the Sierpinski gasket in particular (Fig. 2 (b)) shows unmistakable periodicity. It should be noted that the Weyl ratio graph of the branching-tree (Fig. 2 (c)) is again different from those of other networks, arising from a lack of what we call “looping.” In highly interconnected networks, many cyclic paths exist, allowing a signal to arrive back at a starting vertex by traveling through a series of other vertices. Trees, on the other hand, lack this feature: only one path exists between any two points, helping to create a unique Weyl ratio pattern.

While several cases of slight periodicity could be argued for, this evidence is not definitive enough to indicate self-similarity in the *C. elegans* neural network (Fig. 2 (a)). However, there exists similarity between the Weyl ratio patterns generated by the spectrum of the *C. elegans* neural network, the random network (Fig. 2 (e)), and the rewiring of the Sierpinski Gasket (Fig. 2 (f)). The significance of examining a “rewired” fractal structure will become clear later in this paper. It is important to note that this likeness in Weyl ratio patterns can suggest some structural similarity.

### The Eigen-Projection Method

We replicated the network visualization performed in [25] and extended this technique to our other networks. This was done in Euclidean space via the eigen-projection method explained in [30], similar to those processes described in [31], [32]. This spectral approach to visualizing graphs utilizes the eigenfunctions of degree-normalized Laplacian matrices (see Methods). The eigen-projection method, also known as “plotting in eigenfunction coordinates,” plots the vertices of a graph using the eigenfunctions of its Laplacian matrix as a coordinate basis. See Methods for a more rigorous description.

After embedding each vertex in either 2- or 3-dimensional space, neuronal or network connections were represented with line segments between the appropriate points. In the case of the *C. elegans* diagram, the same color-coding as [25] was utilized: where red represents sensory neurons, green are motor neurons, and blue indicates interneurons. Lastly, points were labeled with the corresponding neuron abbreviations. This was done using a slight variation of the VISUALIZE program used by Chklovski and Varshney, available at [33].

**Figure 3:**
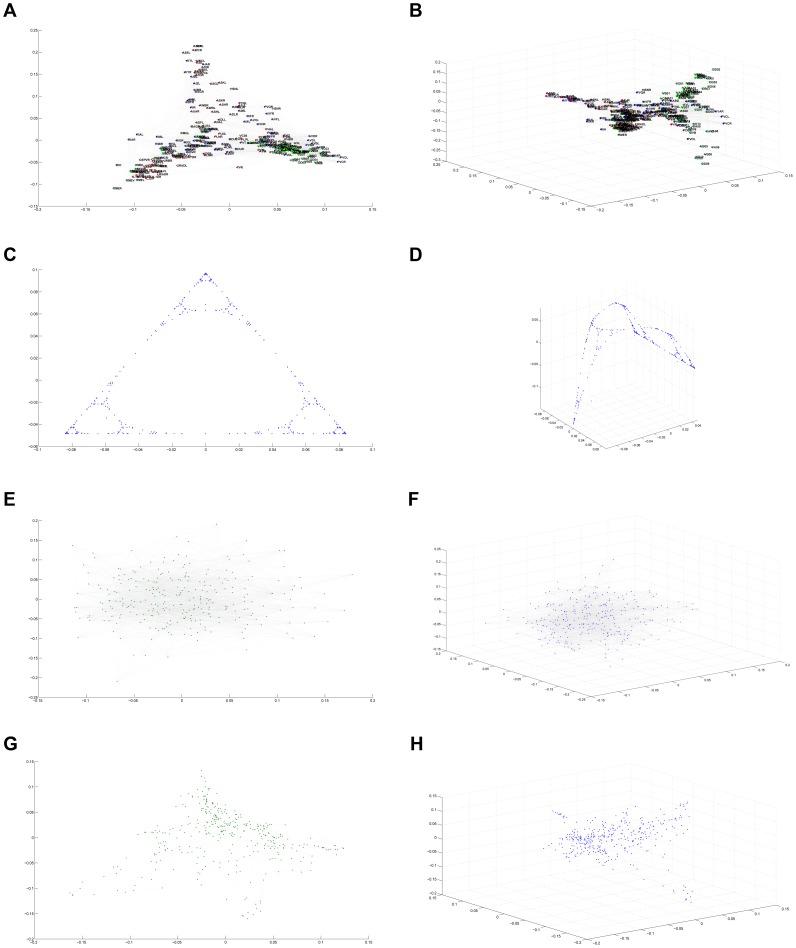
The Eigen Projection Method (a) *C. elegans* neural network, 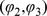 (b) *C. elegans* neural network, 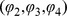 (c) Sierpinski Gasket, Level 5, 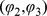 (d) Sierpinski Gasket, Level 5, 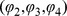 (e) Random Network 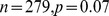, 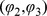 (f) Random Network 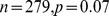, 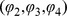 (g) Sierpinski Gasket Rewiring 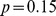, 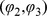 (h) Sierpinski Gasket Rewiring 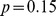, 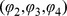.

The eigen-projection visualizations (Figure 3) allow us to make further qualitative distinctions between the *C. elegans* brain and other networks. In support of previous observations, it is again clear that the nematode connectome is not strictly fractal in structure. On the contrary, the eigenfunction graphs of the Sierpinski Gasket once again display characteristics expected of self-similar fractals: a high degree of ordering and self-symmetry. While the eigenvalue counting function and Weyl ratios showed little distinction between the *C. elegans* brain and a random graph, eigen-projections provide differentiation between the two. The random graph appears, as expected, more or less a scatter of points. The *C. elegans* brain, however, shows a definite structure with organized connectivity, suggesting that the *C. elegans* neural network is *not* a randomly connected system of neurons. On the other hand, the *C. elegans* neural network maintains its resemblance to a rewired Sierpinski gasket when plotted in eigenfunction coordinates. While there is no effective way to quantify this heuristic similarity in a relevant manner, it sustains its interest experimentally and continues to suggest the presence of some structural parallels.

The eigen-projections display some of the functional organization of the *C. elegans* neural network. It is clear that the neurons are arranged roughly by neuron type. There is a distinctive cluster of motor neurons (green), a larger sub-component of sensory neurons (red), and interneurons interspersed throughout the network (blue). This indicates that although the brain may not posses the strict self-similarity of a fractal structure, it is indeed highly organized as one would anticipate, developed for entirely functional purposes.

### Small-World Network Properties

We consider two functions defined on graphs: average clustering coefficient and average path length. The clustering coefficient of a vertex 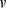, 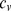, is the probability that any two vertices neighboring 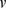 are also connected to each other. The path length between two vertices 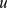 and 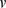 is the shortest path along the graph's edges connecting 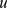 and 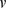 (Note that this path usually travels through a number of other vertices). Using Djisktra's algorithm, it is possible to rigorously determine the shortest path between a given vertex and each other vertex on the graph. By repeating the algorithm for each node on the graph, it is possible to determine the shortest path between each pair of vertices. The average path length, 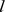, is calculated by finding the arithmetic mean of the shortest paths between each pair of vertices on the graph. Small-world networks are (generally) defined as networks which have a much higher 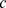 value than random networks, but maintain a value of 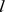 only slightly larger than that of a random network [34].

Small-world networks arise quite often in the natural sciences, as they allow for the efficient transfer of information while maintaining a certain level of complexity. There is a great deal of research which suggests that neural networks possess small-world properties [35], [36]. In fact, [25] and [34] have previously demonstrated that the *C. elegans* neural network is small-world in nature. Our calculations of clustering coefficient and average path length confirm these findings. As Table 1 shows, the *C. elegans* neural network has an average path length only slightly larger than that of its associated random network (see Methods for how these ‘associated random networks’ were constructed). At the same time, the clustering coefficient for *C. elegans* is six times larger than that of its associated random network, meaning the neural network of *C. elegans* satisfies the small-world properties as defined by [34].

**Table 1:** Clustering coefficient and path length.

Graph	Clustering Coefficient	Average Path Length
Sierpinski Gasket, Level 5	0.4495	17.3721
Random(Sierpinski Gasket)	0.0104	5.748
Sierpinski Gasket Rewire 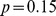	0.2843	7.3833
Random(SG Rewire)	0.0104	5.748
*C. elegans* Neural Network	0.3371	2.5377
Random(*C. elegans* Neural Network)	0.0581	2.3458

This motivated our work with network-rewiring, related to that done by Watts and Strogatz. In [34] they showed that moving connections in an ordered network, with a certain probability 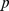, led to some interesting changes in graph structure. Namely, when 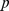 is small, a slight increase in 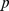 causes a large drop in 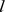 but does not change 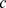 appreciably: the network takes on small-world characteristics. Intuitively this can be explained by the fact that these sparse random connections don't change a graph's strong localized structure, but it becomes easier to travel long distances via these new connections which can span large gaps. It is clear from Table 1 that the Sierpinski Gasket does not possess small-world characteristics. This supports the propositions of [37], which describes the existence of a dichotomy between fractal structures and small world networks. As a result, the Laplacian matrix of a small-world rewiring of the Sierpinski Gasket was included throughout this study. Sporns used a similar method in [18] by generating fractal connections and “rewiring” such networks for small-world properties in studying complexity and self similarity in neuron connectivity.

### Energies and Spacial Variances

**Figure 4:**
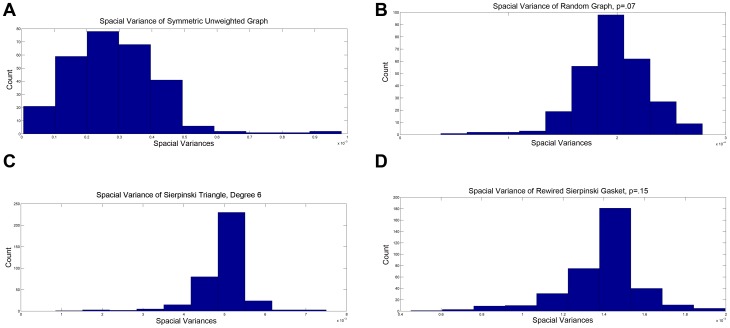
Spacial Variance (a) *C. elegans* neural network (b) Random Graph (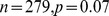) (c) Sierpinski Gasket, Level 5 (d) Sierpinski Gasket Rewiring (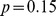).

Using an eigenfunction of a graph's Laplacian, 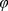, one can calculate a graph energy specific to 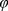. Knowing the resistance between any two vertices and a constant 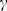, these can be used to calculate the spacial variance of 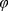 (See Methods). Variance is a measure of how localized a function is, and functions which possess a low spacial variance are said to be localized. In particular, localization occurs when an eigenfunction is approximately zero except for in a a small (localized) region: that is, the eigenfunction takes on most of its values inside a small number of connected regions on the graph. Localized eigenfunctions are known to be present in fractals but not in Euclidean or other smooth spaces [38]. Figure 4 shows distributions of the spacial variance of all eigenfunctions on the graphs used previously.

Of the four graphs considered here, the eigenfunctions of the random network possess the largest spacial variances. As this system was designed to lack general organization, non-localized eigenfunctions were both expected and observed. The eigenfunctions of the Sierpinski Gasket possess both highly concentrated and low-valued spacial variances. Such trends correspond to a high degree of localization, as anticipated in approximations of fractal geometries. Eigenfunctions of the rewired Sierpinski Gasket demonstrate a similar concentration pattern with slightly higher spacial variance, indicating a slightly lower degree of eigenfunction localization.

Of particular interest are the spacial variances of eigenfunctions of the *C. elegans* neural network. Although these spacial variances do not show the same degree of concentration as those of the Sierpinski Gasket, the *values* of these variances are a power of 10 less than those of the fractal network. Whereas spacial variances on eigenfunctions of the Sierpinski Gasket are concentrated around 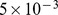 the majority of eigenfunctions of the neural network lie below 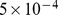. These comparatively lower spacial variances indicate a high level of localization in the eigenfunctions. Such highly localized eigenfunctions can indicate the presence of self-similarity in the network, although further tests would be required to determine the absolute origin of this localization.

In [25] sparsity of eigenfunctions suggests the presence of subcircuits with a specific function. As localization suggests that the value of of an eigenfunction is concentrated on a few vertices, it is reasonable that localized eigenfunction may be used in place of sparse ones. In light of this, the existence of localized eigenfunctions is not entirely unexpected. However [25] looks for sparsity in eigenfunctions of the gap junction network only.

### Conclusions

In this paper we used a variety of graph theoretic and mathematical techniques to probe the structural framework of the *C. elegans* connectome. To better understand results, calculations were also performed on random networks, finite approximations of fractal geometries, and small-world “rewired” graphs. This study confirmed previous results, demonstrating that the neural network exhibits small-world characteristics. Furthermore, the network has highly localized eigenfunctions, which could suggest the presence of self-similar structural motifs. Further research would be required to determine the nature of this localization. Although the *C. elegans* neural network is not random, tree-like, nor fractal in structure, it is certainly highly ordered, aiding in functional efficiency of the system. Although *C. elegans* has proven to be a useful model organism, with a well-defined map of its neural network, this network consists of only 279 nodes. While this makes the system fairly efficient to study computationally, this small number of nodes makes network-analysis rather limited. Although ideally a much larger map would be used, due to the difficulty in determining the exact layout of each neuron in a network, few consistent complete-brain maps exist at this time. In the end, this paper presents a type of analytic “toolbox,” offering many mathematical techniques which can be used to search for structural self-similarity within networks. In particular, the tools presented here can be used to study higher-order brains as more complex neural networks become well-understood.

## Methods

In order to analyze only the framework of the *C. elegans* neural network, we constructed a Laplacian matrix derived form the adjacency matrices in [33]. The network of chemical synapses sends signals in one direction only, resulting in a non-symmetric adjacency matrix, 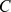. To disregard this directionality, we added this matrix to its own transpose, 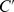, creating a symmetric matrix indexing all chemical connections. We added this matrix to the adjacency matrix of the gap junction system, 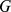 (already symmetric as these connections are bidirectional).
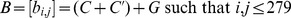
All non-zero entries of this combined matrix, 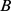, were normalized to be 1, avoiding multiplicity of connection, resulting in matrix 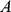.
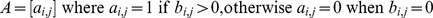
It is then simple to produce a Laplacian matrix, 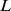, as shown below:
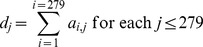
Note that 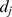 is the degree of each vertex 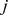. The degree matrix, 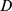, is now defined as:
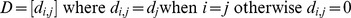
Then the Laplaican matrix, 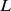, is given by:
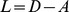



### Generating Random Graphs and Trees

In order to generate a Laplacian matrix representation of the random graphs, we used the following algorithm:

First, fix the number of vertices, 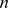, and the probability of connection, 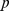, and construct an empty 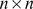 matrix, 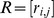.

For each 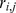 such that 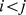 assign a random value 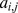 such that 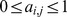 for all 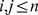. If 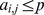 then 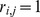, otherwise 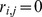.

To produce an adjacency matrix of this graph, 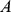, add 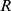 to its own transpose:
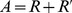
Using this adjacency matrix, construct a Laplacian matrix using the method described previously.

The algorithm used for producing the Laplacian matrix of a random-branching tree is more involved. Again, fix the number of vertices, 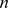, and also specify the maximum number of “children” from any given branch-point, 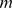. Create an empty 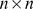 matrix, 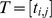


Begin by generating a random integer 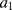 such that 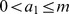, and take 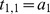. This corresponds to the first vertex having 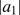 branches. To represent these branches in the matrix, take 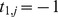 for 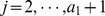 and 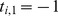 for 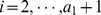.

Next move to all subsequent vertices. Because no “looping” exists in the structure of the tree, each node can only be connected to its parent vertex and its “children” vertices. We take 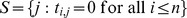 Then 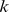, where 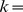
min(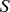) is the smallest-labeled node which does not have a parent vertex, i.e. the first column with all 0 entries corresponds to the first point not yet connected. (Note in the case of vertex 2, 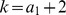). This vertex 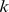 is the first “offspring” from the next branch-point.

Now, as above, for each remaining vertex 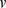 we choose another random integer, 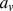, such that 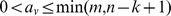 and take 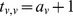. (Note that vertex 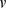 has 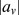 children, however 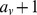 is the degree of node 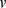, taking into account its parent-connection). To represent the “offspring” branches of this vertex 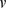, use the following:
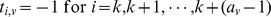
and
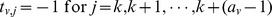
Use min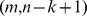 when choosing 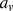 to avoid adding more vertices than the 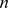 which was originally fixed.

### The Eigenvalue Counting Function and Weyl Ratios

For a given graph Laplacian matrix, 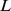, the eigenvalue counting function, 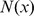 is a cumulative frequency function on the spectrum of the matrix where:
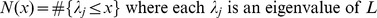
The growth of 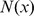 is approximately 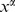, thus the relevant portion of each graph, when using a logarithmic scale, appears linear. A linear regression was found for each relevant interval, and the slope, 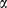, calculated. Using this 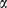, we plotted the Weyl ratio, 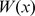, such that:
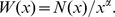
These Weyl ratios allow us to examine the spectrum of each matrix, looking for elements such as symmetry and periodicity [29].

### Normalizing a Laplacian Matrix

**Figure 5:**
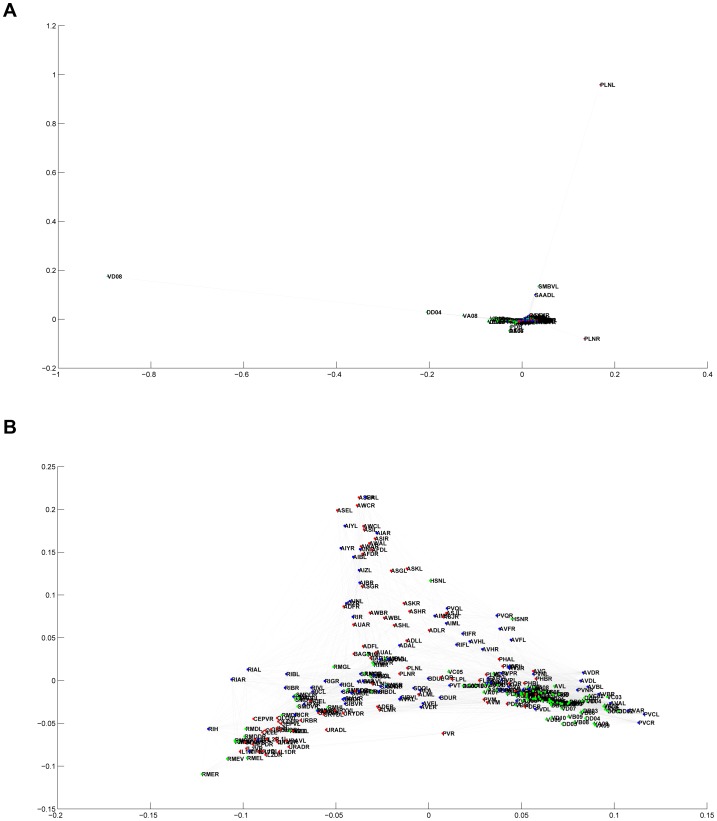
Normalizing the Laplacian *C. elegans* neural network: (a) Un-normalized Laplacian, 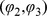 (b) Normalized Laplacian 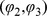.

We used two different forms of the graph-Laplacian matrix: the standard Laplacian and the degree-normalized Laplacian. In the case of eigen-projections, we utilize the degree-normalized matrix. We define the degree matrix, 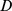, as before: a diagonal matrix whose non-diagonal elements are 0, and each entry 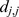 is the degree of the 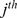 vertex. Using both the standard Laplacian, 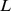 and its corresponding degree matrix, 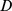, produce the degree-normalized Laplacian, 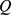:
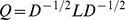
Using the degree-normalized Laplacian has many aesthetic advantages, as shown in Figure 5. The normalized matrix also has all eigenvalues 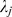 such that 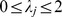.

### Graphing in Eigenfunction Coordinates

We found all eigenvalues, 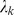, and their corresponding eigenfunctions, 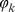, for each matrix. Given two eigenfunctions 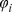 and 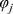, (such that 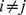) we then plotted the ordered pair 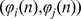 for each 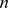 from 1 to 279, as described in [39]. The first eigenvalue of any Laplacian matrix is always 0, corresponding to a constant eigenfunction. Thus we only consider 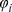 and 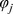 with 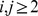. Edges were then added between points to represent relevant connections, and the same color-coding as [25] was used. The same process was then repeated in three dimensions, plotting 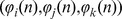 for some 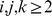, such that 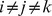.

### Clustering Coefficient

The clustering coefficient measures the probability that two neighbors of a given vertex are also connected to one another. For a graph 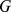 and a given vertex 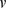, let 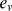 denote the number of connections that exist between the neighbors of 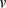. Take 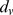 as the number of neighbors of 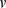 (the degree of vertex 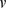). Then the clustering coefficient of vertex 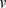, 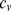, is given by:
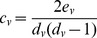
Note that total number of possible connections among neighbors of 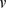 is 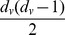.

For a graph 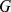 with 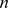 vertices, the average clustering coefficient, 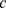, is defined as:
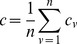



### Generating a Related Random Graph for Small-World Analysis

In order to analyze our networks for small-world properties, it was useful to compare these graphs to those of similar networks with randomly assigned edges. Small-world networks are nearly as well-connected as random graphs, but possess a well-localized structure. We developed the following algorithm for this process:

For a graph 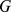 with 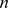 vertices, let 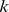 be the number of edges on 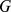. The average number of edges per vertex is 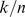. Furthermore, the probability that any two random vertices are connected, 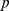, is given by the number of existing connections divided by the total possible connections:
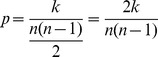
Next we generate a random graph, 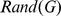, with 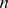 vertices and a 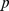 probability of connection between two vertices (See Methods). We then compute 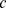 and 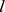 for 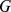 and 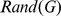.

### Graph Rewiring

First number each vertex in 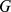 from 1 to 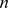, the total number of vertices. If there is a connection between vertices 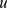 and 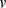 in 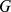, we generate a random number between 0 and 1. If this random number is less than a given probability 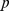, then the connection will be rewired. Without loss of generality assume 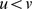. We then fix the connection to vertex 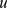, and move the connection to another vertex, 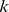, such that 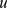 and 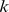 are now connected whereas they were not previously.

### Graph Energy

For a graph 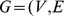) where 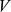 is the set of vertices and 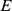 is the set of edges, one can define an arbitrary scalar function 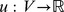. The energy of 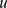 associated with 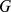, 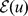 is defined as:
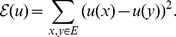
We analyzed the energies of the Laplacian matrix eigenfunctions, thus 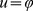.

### Spacial Variance

In order to discuss spacial variance, we must first define the resistance between two vertices on a graph. Let 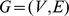 be a graph and 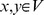. Then the resistance between 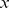 and 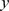, 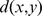, is given by:
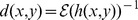
Where 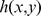 is a harmonic function defined as follows:

Let 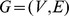 be a graph and 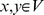. Then the harmonic function corresponding to 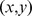 is a scalar function 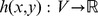 such that:

1. 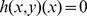



2. 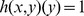

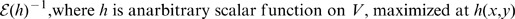
.

Finding the harmonic function is equivalent to finding a vector 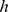 such that 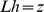, where 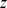 is a vector whose entries are all 0 except for those entries corresponding to 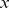 and 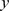. This is analogous to what “harmonic” means in Euclidean space. This changes the maximization problem in condition 3 to solving a system of linear equations.

Using these we can now define the spacial variance of a graph. Again, let 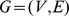 be a graph with 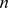 vertices and 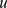 be a scalar function on 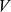. Let 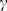 be a constant. Then the 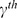 spacial variance of 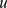 over 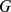 is given by:
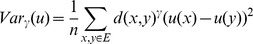
In this paper, the spacial variances of eigenfunctions of Laplacian matrices were evaluated at 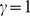.
